# MetamORF: a repository of unique short open reading frames identified by both experimental and computational approaches for gene and metagene analyses

**DOI:** 10.1093/database/baab032

**Published:** 2021-06-22

**Authors:** Sebastien A Choteau, Audrey Wagner, Philippe Pierre, Lionel Spinelli, Christine Brun

**Affiliations:** Aix-Marseille University, INSERM, TAGC, Turing Centre for Living Systems, 163 Avenue de Luminy, Marseille 13009, France; Aix-Marseille University, INSERM, CNRS, CIML, Turing Centre for Living Systems, 163 Avenue de Luminy, Marseille 13009, France; Aix-Marseille University, INSERM, TAGC, Turing Centre for Living Systems, 163 Avenue de Luminy, Marseille 13009, France; Aix-Marseille University, INSERM, CNRS, CIML, Turing Centre for Living Systems, 163 Avenue de Luminy, Marseille 13009, France; Department of Medical Sciences, Institute for Research in Biomedicine (iBiMED) and Ilidio Pinho Foundation, University of Aveiro, Aveiro 3810-193, Portugal; Shanghai Institute of Immunology, School of Medicine, Shanghai Jiao Tong University, Shanghai, China; Aix-Marseille University, INSERM, TAGC, Turing Centre for Living Systems, 163 Avenue de Luminy, Marseille 13009, France; Aix-Marseille University, INSERM, CNRS, CIML, Turing Centre for Living Systems, 163 Avenue de Luminy, Marseille 13009, France; Aix-Marseille University, INSERM, TAGC, Turing Centre for Living Systems, 163 Avenue de Luminy, Marseille 13009, France; CNRS, 31 Chemin Joseph Aiguier, Marseille 13009, France

## Abstract

The development of high-throughput technologies revealed the existence of non-canonical short open reading frames (sORFs) on most eukaryotic ribonucleic acids. They are ubiquitous genetic elements conserved across species and suspected to be involved in numerous cellular processes. MetamORF (https://metamorf.hb.univ-amu.fr/) aims to provide a repository of unique sORFs identified in the human and mouse genomes with both experimental and computational approaches. By gathering publicly available sORF data, normalizing them and summarizing redundant information, we were able to identify a total of 1 162 675 unique sORFs. Despite the usual characterization of ORFs as short, upstream or downstream, there is currently no clear consensus regarding the definition of these categories. Thus, the data have been reprocessed using a normalized nomenclature. MetamORF enables new analyses at locus, gene, transcript and ORF levels, which should offer the possibility to address new questions regarding sORF functions in the future. The repository is available through an user-friendly web interface, allowing easy browsing, visualization, filtering over multiple criteria and export possibilities. sORFs can be searched starting from a gene, a transcript and an ORF ID, looking in a genome area or browsing the whole repository for a species. The database content has also been made available through track hubs at UCSC Genome Browser. Finally, we demonstrated an enrichment of genes harboring upstream ORFs among genes expressed in response to reticular stress.

**Database URL**  https://metamorf.hb.univ-amu.fr/

## Introduction

Short open reading frames (sORFs) are usually defined as sequences delimited by a start codon and a stop codon and potentially translatable into proteins of <100 amino acids (1–8). They are present in all classes of transcripts [including presumptive long non-coding ribonucleic acids (lncRNAs)] and have been identified in most eukaryotic RNAs (2, 5, 8–15). In addition, their sequence often begins with a non-canonical start codon (8). Consequently, they have long been overlooked, and interest in their possible regulatory functions has only raised recently with the advent of the ribosome profiling method that strongly suggests their translation (1, 3, 5, 6, 16–22).

**Figure 1.**
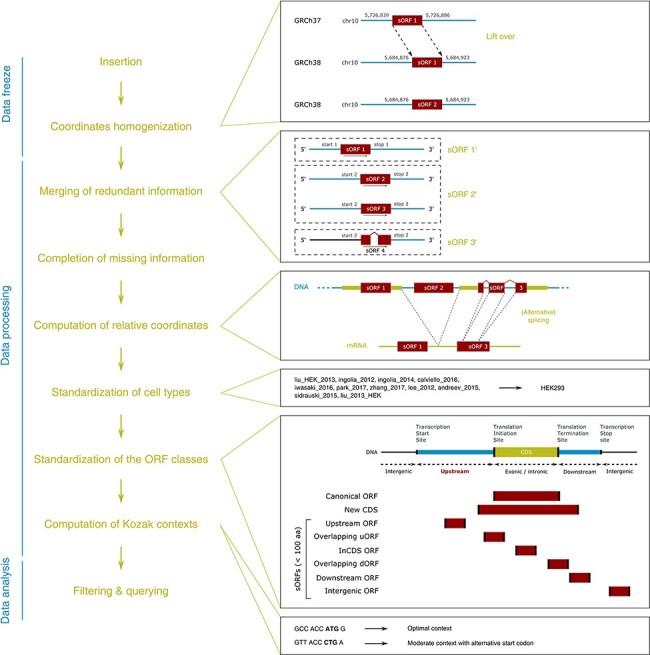
MetamORF pipeline. This figure represents the workflow used to build MetamORF. First, the data from the sources selected have been inserted into the database, and the absolute genomic coordinates have been homogenized from their original annotation version to the most recent version (GRCh38 or GRCm38). Then, the redundant information, i.e. the entries describing the same ORFs (same start, stop and splicing), has been merged, allowing to get one single and unique entries for each ORF detected on the human and mouse genomes. The missing information (sequences and transcript biotypes) has been downloaded from Ensembl, and the ORF relative coordinates have been computed. Finally, the cell types and ORF classes have been normalized, and the Kozak contexts have been computed using the sequences flanking the start codons.

Several sORF categories have been defined according to their location on RNAs (Figure 1). For instance, upstream ORFs (uORFs) are located in the 5ʹ untranslated regions (5ʹ UTRs) of messenger RNAs (mRNAs) and have been defined as sORFs whose start codon precedes the main coding sequence (CDS; 6, 8, 17, 18, 23). They are conserved across species (5, 6, 11, 21, 24), but less conserved than canonical protein-coding ORFs (25). To date, uORFs have been essentially reported as gene-expression *cis*-regulatory elements that regulate the efficiency of translation initiation of the main CDS, notably alleviating the repression of translation during cellular stress (13, 17, 18, 20, 23, 26). Moreover, the discovery of uORF-encoded peptides, and more generally sORF-encoded peptides, led to the assumption that they may also play functional roles in *trans* (2–4, 7, 9, 10, 18, 24, 27–30), for instance as ligands of major histocompatibility complex molecules (12, 22, 23). Very interestingly, uORF-encoded peptides have also been shown to form protein complexes with the protein encoded by the main CDS of the same mRNA (31), and it has been suggested that polycistronic sequences may exist in eukaryotes (24, 31). Furthermore, given the increasing evidence on the regulatory functions of peptides encoded by sORFs located within mRNAs, introns of pre-mRNAs, lncRNAs and primary transcripts of microRNAs or ribosomal RNAs (2, 8–15, 26), there is an urgent need to study sORFs (i) individually and (ii) at the whole proteome scale. Indeed, the latter should reveal important features of sORFs, thus enabling the characterization and the identification of their functions. However, the fact that (i) the publicly available data are scattered across different databases and (ii) datasets are aligned on different genome builds, differently annotated and formatted, calls for an uniformed resource where each sORF is individually described. With this in mind, we have built a resource database of publicly available sORFs identified in the human and mouse genomes, by gathering information from computational predictions and Ribo-seq and proteomic experiments. The curation of data, their homogenization in order to merge the redundant information into unique entries, the completion and computation of missing information (e.g. sequences and Kozak contexts) and the re-annotation of sORF classes represent the added value of this database. Notably, this enables the analyses at locus, gene, transcript and ORF levels. In this work, we propose (i) a pipeline to regularly update the content of the database in a reproducible manner, (ii) a database content that can be fully downloaded for custom computational analyses and (iii) an user-friendly web interface to ease data access to biologists.

## Material and methods

### MetamORF pipeline and database development

#### Inclusion criteria for publicly available sORF-related data

A total of 18 data sources, either *Homo. sapiens* and *Mus. musculus* original datasets or re-processed publicly available sORFs repositories, have been considered for inclusion in our database (Supplementary Table S1) (5, 7, 11, 12, 14, 15, 17–22, 32–37). These data sources provide results from computational predictions, Ribo-seq experiment analyses and mass spectrometry (proteomics/proteogenomics) analyses. The data sources not providing the absolute genomic coordinates of the ORF start and stop codons (5, 17, 20, 32–34) or fully included in another data source considered here (21) have been discarded. Databases that did not allow export of their content in a single file or automating the download of all the files from their website have also been discarded (19, 35). Despite their short size, it has been noticed that sORFs can be spliced. Theoretical lengths of the ORFs have been computed as the distance between the start and stop codons, eventually removing the intron length(s) when information about ORF splicing was provided. Due to splicing, the theoretical length and the one reported by the data source may be different. Data sources harboring this difference for >95% of their entries were discarded as this indicates the splicing information was missing (10). Finally, data sources for which we were not able to perform this assessment as they were not providing information regarding (i) the splicing of the ORF and (ii) ORF length (15, 36) have not been included as well. Hence, the database has been made by collecting data from six distinct sources (Table 1), including either original datasets (Table 1 and Supplementary Table S2) (11, 12, 14, 18, 22) or reprocessed data (37), and discarding 12 of them (Supplementary Table S1). Notably, we have included data from sORFs.org (37), considered as the main and most comprehensive repository of sORFs identified by genome-wide translation profiling (Ribo-seq), that currently integrates re-processed data from 73 original publications.

**Table 1.** Information about the data sources used to build MetamORF

Publication	DOI
Mackowiak *et al.*, 2015, *Genome Biol.* (11)	10.1186/s13059-015-0742-x
Erhard *et al.*, 2018, *Nat. Methods* (22)	10.1038/nmeth.4631
Johnstone *et al.*, 2016, *EMBO J.* (18)	10.15252/embj.201592759
Laumont *et al.*, 2016, *Nat. Commun.* (12)	10.1038/ncomms10238
Samandi *et al.*, 2017, *eLife* (14)	10.7554/eLife.27860
Olexiouk *et al.*, 2018, *Nucleic Acids Res.* (37)	10.1093/nar/gkx1130

See Supplementary Table S1 for more information about these data sources.

For each of these sources, a set of features essential to properly characterize the sORFs, related to their location, length, sequences, environmental signatures and cell types (i.e. cell lines, tissues or organs) in which they are expressed, have been collected (see Table 2 for a full list of features considered for inclusion). When it was not provided by the source, the symbol of the gene related to the sORF was recovered using the transcript identifier (ID, if provided) or searching for the gene(s) or ncRNA(s) overlapping with the sORF coordinates in the original annotation version by querying Ensembl databases (38) in their appropriate versions (v74, 75, 76, 80, 90) with pyensembl (v1.8.5, https://github.com/openvax/pyensembl). In addition to these features, information regarding the transcript(s) harboring the ORFs have been collected from the data sources when available. This is of particular interest as some ORF features, such as the ORF class, may depend on the transcript they are located in (e.g. an ORF may be located in the 5ʹ UTR of a transcript and be overlapping with the CDS of another transcript). Finally, 3 379 219 and 2 066 627 entries from these six data sources have been collected and inserted in MetamORF for *H. sapiens* and *M. musculus*, respectively (Table 3).

**Table 2.** Features allowing to characterize the sORFs

Family	Feature	Details
Location	Chromosome	The chromosome or scaffold on which the ORF is located
	Strand	The strand of the sORF
	ORF start	The absolute genomic coordinates of the start codon (position of the first nucleotide)
	ORF stop	The absolute genomic coordinates of the stop codon (position of the third nucleotide)
	Splicing status	Is the sORF spliced?
	Splicing coordinates	The coordinates of the start and end of each exon constituting the sORF
	Transcript	The name or ID of the transcript(s) related to the sORF (eventually with transcript strand, start and end positions and transcript biotype)
	Gene	The name, symbol, alias or ID of the gene(s) related to the sORF (when not intergenic)
Lengths	Length	The length of the sORF (in nucleotides)
	Putative sPEP length	The length of the (putative) sORF-encoded peptide in amino acids
Category	Category	The category to which the sORF belongs (e.g. upstream or downstream)
Sequence signature	Start codon sequence	The nucleic sequence of the sORF start codon
	Nucleic sequence	The nucleic sequence of the sORF
	Amino acid sequence	The amino acid sequence of the (putative) sORF-encoded peptide
Environmental signature	Kozak context	Does a Kozak context has been identified for the sORF start codon?
Conservation	PhyloCSF score	The PhyloCSF score computed for the sORF
	PhastCons score	The PhastCons score computed for the sORF
Coding potential assessment	FLOSS class and score	The FLOSS class and score computed for the sORF
	ORF score	The ORF score computed for the sORF
Biological context	Cell context	The cellular context in which the sORF has been identified or detected

#### Homogenization of genomic coordinates

As the data sources were providing genomic coordinates from different genome annotation versions (e.g. GRCh38 and GRCh37), all the genomic coordinates registered in our database have been lifted over the latest annotation version (GRCh38 for *H. sapiens* and GRCm38 for *M. musculus*) using pyliftover (v0.4, https://pypi.org/project/pyliftover). The liftover has been considered as failed for an entry if (i) at least one of the coordinates (i.e. start, stop or one of the start or end exon coordinates) was located on a strand different from all the others or (ii) the chromosome of the position changed during the liftover or (iii) the distance (in nucleotides) between the sORF start and stop codons has changed after the liftover. All the entries for which the liftover failed were removed from the database. Based on the previous assumptions, the liftover failed for 709 ORFs (377 failed due to the last criteria) in *H. sapiens* and for none of the *M. musculus* entries (Table 3). The choice of such stringent criteria has been strengthened by the fact that these entries (i) only represent <0.05% of the entries for *H. sapiens* and (ii) are more susceptible to be unreliable entries.

**Table 3.** MetamORF most important statistics

Feature		*H. sapiens*	*M. musculus*
Original data sources	ORFs	1 344 978	1 249 176
	Transcripts	101 597	85 653
	Predicted ORFs for which the transcript is unknown	181 122	213 301
	ORFs detected by Ribo-seq for which the transcript is unknown	79 422	8546
	ORFs detected by MS for which the transcript is unknown	54	0
	ORF to transcript associations	3 379 219	2 066 627
	ORFs predicted	202 309	222 705
	ORFs identified by ribosome profiling	1 142 669	1 026 471
	ORFs identified by MS	166	0
ORFs for which the homogeneization of genomic coordinates failed		709	0
MetamORF database	ORFs	664 771	497 904
	Transcripts	90 406	63 147
	Predicted ORFs for which the transcript is unknown	13 440	14 327
	ORFs detected by Ribo-seq for which the transcript is unknown	71 158	2
	ORFs detected by MS for which the transcript is unknown	48	0
	ORF for which the transcripts are unknown	83 403	14 329
	ORF to transcript associations	729 793	696 785
	ORFs predicted	17 027	14 500
	ORFs identified by ribosome profiling	664 771	497 904
	ORFs identified by MS	147	0
Genes harboring at least 1 sORF		23 767	15 869
ORFs having at least one class annotation (short, upstream)		630 953	497 904

MS: mass spectrometry.

#### Merge of redundant information

As our database aims to provide a repository of unique identified sORFs of the human and mouse genomes, all the redundant entries describing the same sORFs have been merged. In a first step, we identified all the sORF entries for which all the identification features were provided (chromosome, strand, start position, stop position, splicing status and splicing coordinates). sORFs sharing the same feature values were merged. In a second step, we identified all the remaining entries with only partial identification features provided: the chromosome as well as either (i) both the strand and the start positions or (ii) both the strand and the stop positions or (iii) both the start and the stop positions. Those entries were merged to the best matching fully described entries identified in the first step. If no matching fully described entry was found, then the entries were removed. In order to keep track of the number of times a same sORF has been described in the original data sources, the initial number of entries merged together was registered for each sORF.

During this merging, information regarding the transcripts that harbor the sORFs has been registered too. Hence, when several sORFs were merged into one single entry in MetamORF, the resulting new sORF entry was registered as harbored by all the distinct transcripts related to the original entries. After this removal of redundant information, we were finally able to identify 664 771 and 497 904 unique sORFs for *H. sapiens* and *M. musculus,* respectively (Table 3).

It should be noticed that all unique sORF entries generated at this stage have been kept, including the ones describing ORFs longer than 100 amino acids. Entries describing such ORFs may be either coming from data sources that (i) did not remove the ORFs longer than 100 amino acids or (ii) used a higher threshold or (iii) described the ORF as unspliced while it is actually susceptible to be spliced (and thus has a shorter sequence on the transcript than the one expected).

#### Completion of missing information and computation of relative coordinates

In the original data sources, the only information provided (when provided) on the transcripts was the transcript ID. Detailed information was retrieved from Ensembl databases (v90) through their REST API and inserted in our database: (i) the transcript biotype, (ii) the transcript start and end genomic coordinates, (iii) the codon of the canonical CDS (for protein-coding transcripts only) start and stop genomic coordinates and (iv) the full nucleic sequence. In addition, the sequence flanking the start codon (20) has been recovered. As the sORF nucleic and amino acid sequences were not systematically provided by the data sources, these were downloaded from the Ensembl databases using their genomic coordinates.

Moreover, when the transcript ID was available, sORF start and stop relative coordinates have been computed on each of their transcript using AnnotationHub (v2.18.0; 39) and ensembldb (v2.10.2, https://bioconductor.org/packages/release/bioc/html/ensembldb.html) R packages (R v3.6.0).

### Standardization of the cell types and ORF classes

#### Cell types

Original data sources do not use a common thesaurus or ontology to name the cell types (e.g. ‘HFF’ and ‘Human Foreskin Fibroblast’) or use non-biological meaning names (e.g. sORFs.org (37) provides the name of the original publication as a cell type). In order to provide an uniform informative naming, we manually recovered the name of the cell line, tissue or organ used in these datasets and defined an unique name to be used in our database for each cell line, tissue or organ, trying to use the most commonly used nomenclature for cell lines (Supplementary Table S3). In addition, in order to ensure interoperability with other biological resources, we recovered the matching ontology terms from the following ontologies when feasible: the Cell Ontology (40), the Cell Line Ontology (41), the BRENDA Tissue Ontology (42), the Human Cell Atlas Ontology (43), the Foundational Model of the Anatomy Ontology (44), the Ontology for Biomedical Investigations (45), the NCI Thesaurus OBO Edition (46), the Experimental Factor Ontology (47), the BioAssay Ontology (48) and the Ontology for MIRNA Target (49), using the Ontology Lookup Service (EBI) (50) (Supplementary Table S4).

#### ORF classification

Despite the use of a common nomenclature by the wide majority of the scientific community to annotate the open reading frames, based on their size and relative position on their transcript (e.g. short, upstream, downstream and overlapping), no clear consensus about the definitions of these categories nor their names has been defined so far (25). In order to homogenize this information in MetamORF, we created a new annotation of the ORFs using the ORF length, transcript biotype, relative positions and reading frame information when available (see Supplementary Methods). In this annotation, a threshold of 100 amino acids has been used to define the ‘short ORFs’, as this value is the most commonly used for historical reasons (2, 4, 6, 8, 24).

#### Computation of the Kozak contexts

The Kozak motif and context have been regarded as the optimal sequence context to initiate translation in all eukaryotes. We have thus assessed the Kozak context for each sORF, using the criteria defined by Hernández *et al.* (51). Briefly, for each ORF to transcript association, the Kozak context was computed looking for regular expression characterizing an optimal, strong, moderate or weak Kozak context (Supplementary Tables S5 and S6). Kozak-alike contexts were also computed for non-ATG initiated sORFs looking for the same patterns with flexibility regarding nucleotides at +1 to +4 positions.

### MetamORF software and languages

The pipeline used to build MetamORF has been developed using Python (v2.7) with SQLAlchemy ORM (sqlalchemy.org, v1.3.5). The database has been handled using MySQL (mysql.com, v8.0.16). Docker (docker.com, v18.09.3) and Singularity (singularity.lbl.gov, v2.5.1) environments have been used in order to ensure reproducibility and to facilitate deployment on high-performance clusters.

The MetamORF web interface has been developed using the Laravel (laravel.com, v7.14.1) framework with PHP (v7.3.0), JavaScript 9, HTML 5 and CSS 3. The NGINX (v1.17.10) web server and PHP server (v7.3.0) were deployed with Docker (docker.com, v18.09.3) and Docker-compose (v1.24.0) to ensure stability.

### Enrichment analysis

#### Gene lists

The list of genes harboring at least one uORF has been collected from MetamORF as a list of Ensembl identifiers using a SQL query.

The list of ATF4 and CHOP targets identified by ChIP-seq comes from Han *et al.* (52) (available as supplementary data on the editor’s website). Genes congruently and translationally upregulated under endoplasmic reticular (ER) stress have been provided by Guan *et al.* (53) (upon request). As these lists of genes were provided as gene symbols, matching Ensembl IDs have been recovered using the g:Convert tool available on the gProfiler web interface (54).

The universe contains all protein-coding genes annotated at least once in Gene Ontology (55, 56) (downloaded from the g:Profiler web interface on 3 November 2020).

#### Statistics

After discarding genes absent in the universe from the lists, the enrichment analysis was performed using an hypergeometric test with R 3.6.0 (https://www.r-project.org/). A Benjamini–Hochberg correction has been applied to allow for multiple comparisons, and a False Discovery Rate (FDR) threshold of 0.05 has been considered as significant.

## Database content, accessibility and web interface

### A new repository of short ORF-related data

**Figure 2.**
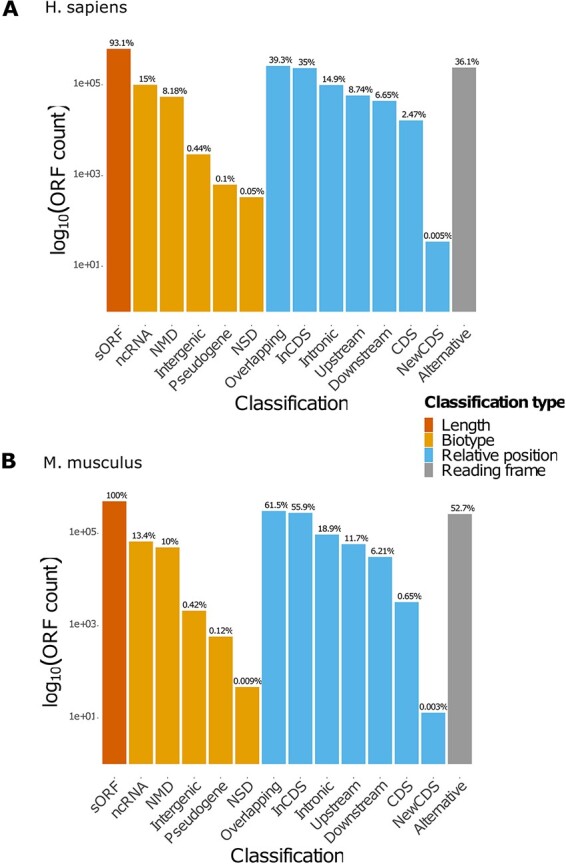
Count of ORFs in each class. The bar plots represent the count of ORFs annotated for each class for (A) *H. sapiens* and (B) *M.* musculus. The percentages displayed over the bars indicate the proportion of ORFs annotated in the class over the total number of ORFs registered in the database for the species. NMD: non-sense-mediated decay; NSD: non-stop decay.

MetamORF describes 664 771 and 497 904 unique ORFs in the human and mouse genomes, respectively, providing at least the information necessary to locate the ORF on the genome, its sequence and the gene it is located on (excepted for intergenic ORFs). Extensive information related to the transcripts is provided for 614 997 (∼93%) and 497 904 (100%) sORFs for the human and mouse genomes, respectively. These features allowed us to classify 630 953 (∼95%) human ORFs and 497 904 (100%) mouse ORFs in at least one class (Table 3, Figure 2, Supplementary Figure S1). Interestingly, it should be noticed that a large proportion (36% and 52% for *H. sapiens* and *M. musculus* respectively) of ORFs are using an alternative frame to the main CDS. In addition, nearly 23% of the ORFs are located on non-coding RNAs for both species.

### User-friendly web interface and genome tracks

**Figure 3.**
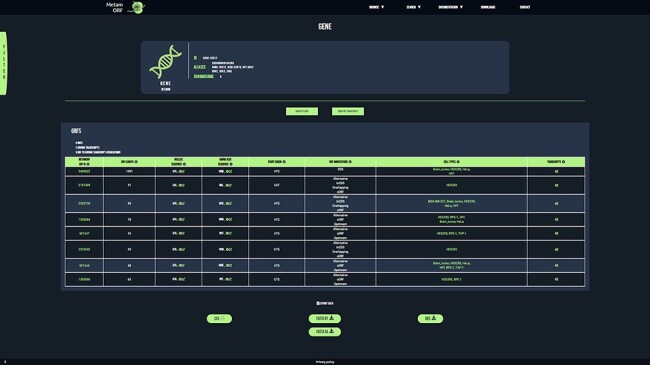
MetamORF gene-centric view. The page displays the transcripts and the ORFs related to *SGK3* gene. A filter has been applied to select exclusively the ORFs detected in HFF, Jurkat, RPE-1, HEK293 or HeLa cells. Other filters may be used and the results can be exported as CSV, FASTA or BED files.

To provide users with a clear, fast and easy-to-use database, MetamORF can be queried through an user-friendly web interface at https://metamorf.hb.univ-amu.fr. A tutorial as well as a documentation page are available online. Briefly, the users may search for sORFs contained in the database starting with a gene symbol (symbol, alias, ID), a transcript ID (ID, name) and an ORF ID or screening a particular genomic area. The data are made accessible through four types of pages: (i) a ‘gene’ page (Figure 3) to allow visualizing information related to all transcripts and sORFs on a gene, (ii) a ‘transcript’ page to allow browsing information related to a transcript gene and all its sORFs, (iii) an ‘ORF’ page to allow fetching information related to all transcripts and gene that harbor the chosen ORF and finally (iv) a ‘locus’ page to allow getting information related to all sORFs located in a particular locus. In addition, the user may also browse across all sORFs related to a species or detected in a particular cell type. It is possible to navigate from one to another page easily to get extensive information about a sORF, a gene or a transcript (Supplementary Figure S2).

In each page, the results can be filtered on (i) the identification method (computational prediction, ribosome profiling or mass spectrometry), (ii) the start codon, (iii) the Kozak context (as previously defined), (iv) the genomic length (defined as the sum of lengths of each exon constituting the ORF), (v) the transcript biotype (according to the Ensembl definitions), (vi) the ORF annotation (as previously defined) and (vii) the cell type (Supplementary Tables S3 and S4).

All results can be exported in an easily parsable format (comma-separated values file, CSV), as well as in FASTA or BED format.

On ORF, transcript and locus pages, a link allowing the user to easily visualize all the ORFs localized in a particular area on the UCSC Genome Browser (57) is proposed. We also implemented genome track hubs, to allow using UCSC Genome Browser advanced options, such as filtering on ORF categories, transcript biotypes, cell types and transcript IDs.

In addition to this user-friendly interface, it is possible to download from the website the content of the full MetamORF database in BED and FASTA formats.

### Using MetamORF to analyze the regulation of integrated stress response

Several studies have reported the role of uORFs in the regulation of the translation during the integrated stress response (ISR) (13, 23, 26, 28). Notably, the mechanism by which the repression of the translation is alleviated under an ER stress has been elucidated for the mammalian transcription factor ATF4, the targets of which are responsible for cell adaptation to stress. Briefly, ATF4 CDS is preceded by two functional uORFs (58), both highly expressed under normal growth and stress conditions. Under the ISR, the small ribosomal subunit is expected to remain bound to the mRNA, scan through the uORF2 and acquire the eIF2•GTP•Met-tRNA_i_^Met^ and the large ribosomal subunit in time for initiation at the start codon of the CDS, a phenomenon known as ‘leaky scanning’. In addition, it has been also suggested that the translation of the CDS under stress may result from the ‘re-initiation’, a model in which the large ribosomal subunit and the initiation complex are recruited by the small subunit right after the termination of the translation of the uORF2, allowing thus the initiation at the CDS start codon. Both events are nevertheless technically difficult to distinguish and the exact process remains debated. Hence, assuming the presence of one uORF is sufficient to regulate the translation of the CDS (20), are targets of ATF4 and CHOP (another transcription factor activated upon stress) more likely to harbor uORFs than other genes? Are genes translationally or congruently upregulated during an ER stress, enriched in genes harboring uORFs? To answer these questions, we performed enrichment analyses, getting the list of genes harboring uORFs by querying MetamORF, and using the published lists of target genes of ATF4 and CHOP identified by ChIP-seq (Supplementary Table S7). We demonstrated that ATF4 and CHOP targets as well as genes upregulated under an ER stress are more likely to harbor uORFs than expected by chance (OR_ATF4_ = 2.40, pval = 2.76.10^−17^ and OR_CHOP_ = 2.34, pval = 2.50.10^−11^, respectively; Table 4). This suggests that the translation of these genes is likely to be under the control of uORFs, as it has been experimentally shown for PPP1R15A and PPP1R15B (23), two well-known targets of ATF4.

**Table 4.** Enrichment analysis

Gene list^a^	List size	Genes harboring uORFs	Intersection size	Universe^a^ size	FDR	Odds ratio
ATF4 targets	392	8863	256	19 985	5.52.10^−17^	2.40
CHOP targets	256	8863	166	19 985	3.34.10^−11^	2.34
Genes congruently upregulated	484	8863	268	19 985	5.41.10^−7^	1.57
Genes transitionally upregulated	1068	8863	736	19 985	1.21.10^−61^	2.94

a See Supplementary Table S7 for more information about the gene lists.

## Discussion and conclusion

MetamORF contains data about 1 162 675 unique sORFs for the human and mouse genomes identified by both experimental and computational approaches. While the Ribo-seq is considered by most as the ‘gold standard’ method to identify sORFs experimentally, the added value of predictive computational approaches, proteomics and peptidomics to characterize such biological sequences remains certain. Because these technologies are offering complementary information at genomic, transcriptomic and proteomic scales, we decided to include data from both experimental and computational experiments in our database. Nevertheless, data coming from distinct data sources may be difficult to compare, in particular because they are not necessarily using the same genome annotation and definitions of ORF classes and Kozak contexts, for instance. By homogenizing this information, MetamORF offers the possibility to compare datasets coming from different sources. We noticed that information regarding the Kozak context is missing most of the time, and start flanking sequences are usually not provided. Hence, MetamORF provides a new interesting set of information. It is noteworthy that we discarded 12 of 18 datasets because they lack crucial information regarding their integration into MetamORF. Although this is a rather drastic method, this is performed for the sake of data quality. In these conditions, the confidence in the data and the reliability in the existence of the sORF of interest can be assessed by the number of original experiments that identify the sORF (column ‘EXP. COUNT’ in the tables of the web interface). It is noteworthy that >97% of the unique ORF entries registered in MetamORF have been identified by at least one experimental method.

It should be noticed that a large amount (∼80%) of the sORFs contained in our database have been described in the sORFs.org repository (37). Despite being the most prominent sORF database and offering the community data processed in a normalized way using their own workflow, sORFs.org does not provide metagene analyses (1). In addition, such analysis is made difficult by the absence of gene names and transcriptomic coordinates as well as the high redundancy of information contained in the sORF.org database (37), issues that we addressed with MetamORF. It is noteworthy that another sORF resource, namely OpenProt (59), does not contain ORFs shorter than 30 amino acids, whereas in MetamORF, sORFs of such size represent ∼50% of the dataset. Of note, 54% of them have been detected in at least two data sources, therefore reinforcing their probability of existence. Hence, in comparison with existing resources (Supplementary Table S8), MetamORF is complementary and allows analyses at ORF, transcript, gene and locus levels. In addition, it opens the possibility of studying sORFs as a group, at a global scale.

The resource is accessible at https://metamorf.hb.univ-amu.fr and provides an intuitive querying interface to enable wet-laboratory researchers to easily question this large set of information. The web interface comes with advanced filters, notably on computed ORF classes, ORF start codons, identification methods, Kozak contexts and cell contexts. Such filters should help end-user biologists without computational skills to identify and collect information about the sORFs important for their topic of interest. Moreover, the implementation of MetamORF content in track hubs allows both quick and advanced visualization of data through the UCSC Genome Browser. Finally, the database content may be exported in various convenient formats widely used by the scientific community (e.g. FASTA and BED).

We believe that MetamORF is of interest not only to bioinformaticians working on short ORFs but also to a wider community, including any biologist who may benefit from knowledge regarding the sORFs located on their gene, transcript or region of interest. As ribosome profiling becomes more appreciated and proteomics starts allowing accurate identification of short peptides, new data describing sORFs in various conditions will be published in the next years, and our database is expected to grow accordingly. In particular, the next release of MetamORF is expected to include data describing the sORFs of other organisms such as *Drosophila melanogaster*. As a conclusion, we believe that MetamORF should help to address new questions in the future, in particular regarding the regulatory functions of the sORFs as well as the functions of the short peptides they may encode.

## Supplementary Material

baab032_Supp

## Data Availability

Data sources are available on the editor’s website or using the links provided in their original publications. The source code used to create the database and the full technical documentation (source code documentation, manual, database structure and dockerfiles) are available on GitHub (https://github.com/TAGC-NetworkBiology/MetamORF). Full content of the database can be downloaded in BED and FASTA formats from the MetamORF website, and up-to-date version of track hubs may be downloaded and/or used with your favorite genome browser from the link https://metamorf.hb.univ-amu.fr/hubDirectory/hub.txt. The dump of the database is available on request.
