# Supplementary material for: MetamORF: a repository of unique short open reading frames identified by both experimental and computational approaches for gene and metagene analyses
Source: Database (Oxford). 2021 Jun 22;2021:baab032. doi: 10.1093/database/baab032 (PMC8218702; doi:10.1093/database/baab032)
Supplement: baab032_Supp [file baab032_supp.zip › supp_data.pdf]

Supplementary methods

ORF classification

- ORF annotations based on length
  - sORF (short ORF): The ORF sequence is shorter than 100 amino acids (stop codon and intron excluded).
- ORF annotations based on the transcript biotype
  - Intergenic: The ORF is located on a 'Long intergenic ncRNA' or 'lincRNA' biotype.
  - ncRNA: The ORF is located on a 'Non coding', 'ncRNA', 'Processed transcript', 'processed_transcript', 'Long non-coding RNA', 'lncRNA', '3' overlapping ncRNA', 'Macro lncRNA', 'Long intergenic ncRNA', 'lincRNA', 'miRNA', 'miscRNA', 'piRNA', 'rRNA', 'siRNA', 'snRNA', 'snoRNA', 'tRNA', or 'vaultRNA' biotype.
  - Pseudogene: The ORF is located on a 'Pseudogene', 'IG pseudogene', 'Polymorphic pseudogene', 'Processed pseudogene', 'processed_pseudogene', 'Transcribed pseudogene', 'transcribed_processed_pseudogene', 'transcribed_unprocessed_pseudogene', 'unprocessed_pseudogene', 'transcribed_unitary_pseudogene', 'Translated pseudogene', 'Unitary pseudogene', 'unitary_pseudogene' or 'Unprocessed pseudogene' biotype.
  - NMD: The ORF is located on a 'Non sense mediated decay', 'NMD', 'nonsense_mediated_decay' or 'non_stop_decay' biotype.
  - Readthrough: The ORF is located on a 'Readthrough' or 'Stop codon readthrough' biotype.
- ORF annotations based on the relative position
  - Upstream: The start codon of the ORF is located upstream of the CDS start codon and the stop codon of the ORF is located upstream of the CDS stop codon. Note that if both the start and the stop codons are the same for the ORF than the CDS, then the ORF is annotated CDS instead.
  - Downstream:
    - The start codon of the ORF is located downstream of the CDS start codon and the stop codon of the ORF is located downstream of the CDS stop codon. Note that if both the start and the stop codons are the same for the ORF than the CDS, then the ORF is annotated CDS instead, or
    - The ORF is located on a '3'overlapping ncRNA' biotype.
  - Overlapping:
    - The start codon of the ORF is located upstream of the CDS start codon and the stop codon of the ORF is located downstream of the CDS start codon and upstream of the CDS stop codon, or
    - The start codon of the ORF is located downstream of the CDS start codon and upstream of the CDS stop codon and the stop codon of the ORF is located downstream of the CDS stop codon or
    - The ORF is located on an 'Antisense' biotype.
  - Intronic: The ORF is located on a 'retained_intron', 'sense_intronic' or 'sense_overlapping' biotype.
  - InCDS: The start codon of the ORF is located downstream of the CDS start codon and the stop codon of the ORF is located upstream of the CDS stop codon. Note that if both the start and the stop codons are the same for the ORF than the CDS, then the ORF is annotated CDS instead.
  - CDS: The start codon of the ORF is the same than the CDS start codon and the stop codon of the ORF is the same than the CDS stop codon.
  - NewCDS: The start codon of the ORF is located upstream of the CDS start codon and the stop codon of the ORF is located downstream of the CDS stop codon. Note that if both the start and the stop codons are the same for the ORF than the CDS, then the ORF is annotated CDS instead.
- ORF annotations based on the reading frame
  - Alternative: The ORF start is located on a different frame than the CDS start codon (i.e. the distances in bp between the first nucleotide of the ORF start and the first nucleotide of the CDS start is not a multiple of three).

- ORF annotations based on the strand
    - Opposite: The ORF is located on the opposite strand of its transcript.

Supp. Table S1 | Data sources description.

Publication	DOI	Database / Source description	Included	Criteria of exclusion if not included
Andreev et al., 2018, eLife (1)	10.7554/eLife.32563		No	ORF start and stop genomic absolute coordinates missing
Rodriguez et al., 2019, BMC Genomics (2)	10.1101/412106		No	ORF start and stop genomic absolute coordinates missing
Sharipov et al., 2014, Virtual Biology (3)	10.12704/vb/e18	RiboSeqDB	No	ORF start and stop genomic absolute coordinates missing
Evans et al., 2012, Nat. Methods (4)	10.1038/nmeth.2227	PITDB	No	ORF start and stop genomic absolute coordinates missing
Chew et al., 2016, Nat. Commun. (5)	10.1038/ncomms11663		No	ORF start and stop genomic absolute coordinates missing
Wethmar et al., 2014, Nucl. Ac. Res. (6)	10.1093/nar/gkt952	uORFdb	No	ORF start and stop genomic absolute coordinates missing
Fields et al., 2015, Mol. Cell (7)	10.1016/j.molcel.2015.11.013		No	Dataset included in sORFs.org
Liu et al., 2018, Nucl. Ac. Res. (8)	10.1093/nar/gkx1034	TranslatomeDB	No	Automated download of numerous files impossible
Wang et al., 2019, Nucl. Ac. Res. (9)	10.1093/nar/gky978	RPFdb	No	Automated download of numerous files impossible

Hao et al., 2018, Brief Bioinform. (10)	10.1093/bib/bbx005	smPROT	No	ORF theoretical length different from the provided ORF length for more than 95% of the entries, suggesting unregistered splicing
Lee et al., 2012, Proc. Natl. Acad. Sci. USA (11)	10.1073/pnas.1207846109	TISdb. Files downloaded from “download” section.	No	No information provided about the splicing neither about the ORF length
McGillivray et al., 2018, Nucl. Ac. Res. (12)	10.1093/nar/gky188	Supplementary tables S3, S4, S6-S14	No	No information provided about the splicing neither about the ORF length
Mackowiak et al., 2015, Genome Biol. (13)	10.1186/s13059-015-0742-x	Additional file 2: Table S1. All sORF information for human	Yes	
Erhard et al., 2018, Nat. Meth. (14)	10.1038/nmeth.4631	Supplementary Table 3: Identified ORFs (Union of all ORFs detected either by PRICE, RP-BP or ORF-RATER, or contained in the annotation (Ensembl V75))	Yes	
Johnstone et al., 2016, EMBO (15)	10.15252/embj.201592759	Dataset EV2: Location and translation data for all analyzed transcripts and ORFs in human	Yes	
Laumont et al., 2016, Nat. Commun. (16)	10.1038/ncomms10238	Supplementary Data 2: List of all cryptic MAPs detected in subject 1. Table presenting the genomic and proteomic features of all cryptic MAPs	Yes	

Samandi et al., 2017, eLife (17)	10.7554/eLife.27860	*Homo sapiens* alternative protein predictions based on RefSeq GRCh38 (hg38) based on assembly GCF_000001405.26. Release date 01/01/2016 (tsv file).<br>*Mus musculus* alternative protein predictions based on annotation version GRCm38. Release date 01/01/2016 (tsv file).	Yes
Olexiouk et al., 2018, Nucl. Ac. Res. (18)	10.1093/nar/gkx1130	sORFs.org. Download full database content from their website	Yes

Supp. Table S2 | Date of download of the data sources and cross-references

File / data source	Date of download
HGNC cross-references for *H. sapiens*	08/06/2020
NCBI cross-references for *M. musculus*	08/06/2020
sORFs.org - *H. sapiens*	08/06/2020
Erhard *et al.*, 2018 - *H. sapiens (14)*	04/01/2019
Johnstone *et al.*, 2016 - *H. sapiens (15)*	04/01/2019
Laumont *et al.*, 2016 - *H. sapiens (16)*	04/01/2019
Mackowiak *et al.*, 2015 - *H. sapiens (13)*	20/03/2019
Samandi *et al.*, 2017 - *H. sapiens (17)*	04/01/2019
Johnstone *et al.*, 2016 - *M. musculus (15)*	04/01/2019
sORFs.org - *M. musculus*	08/06/2020
Mackowiak *et al.*, 2015 - *M. musculus (13)*	20/03/2019
Samandi *et al.*, 2017 - *M. musculus (17)*	04/01/2019

Supp. Table S3 | Homogenization of cell types

Species	Name of the original cell type (as provided by the data source)	Cell type name used in MetamORF
H. sapiens	loayza_puch_2013	BJ
	rooijers_2013	BJ
	ji_BJ_2015	BJ
	B cells	B_cell
	mills_2016	Blood
	gonzalez_2014	Brain
	Human brain tumor	Brain_tumor
	ji_breast_2015	Breast
	loayza_puch_2016	Breast_tumor
	jakobsson_2017	HAP1
	crappe_2014	HCT116
	lee_2012	HEK293
	andreev_2015	HEK293
	sidrauski_2015	HEK293
	liu_2013_HEK	HEK293
	liu_HEK_2013	HEK293
	ingolia_2012	HEK293
	ingolia_2014	HEK293
	calviello_2016	HEK293
	iwasaki_2016	HEK293
	park_2017	HEK293
	zhang_2017	HEK293
	eichorn_2014	HEK293T
	jan_2014	HEK293T
	Primary human foreskin fibroblasts (HFFs)	HFF
	Primary human fibroblast (HFF)	HFF
	rutkowski_2015	HFF
	wang_2015	HeLa
	niu_2014	HeLa
	yoon_2014	HeLa
	liu_2013_HeLa	HeLa
	liu_Hela_2013	HeLa
	stumpf_2013	HeLa

	park_2016	
	zur_2016	
	shi_2017	
	werner_2015	hES
	xu_2016	
	gawron_2016	Jurkat
	cenik_2015	LCL
	Loayza_Puch_2016	MCF7
	rubio_2014	MDA-MB-231
	wiita_2013	MM1S
	su_2015	Monocyte
	grow_2015	NCCIT
	tanenbaum_2015	RPE-1
	tirosh_2015	
	wein_2014	Skeletal_muscle
	fritsch_2012	THP-1
	stern_ginossar_2012	
	elkon_2015	U2OS
	malecki_2017	Flp-In_T-REx-293
M. musculus	eichorn_3t3_2014	3T3
	jovanovic_2015	BMDC
	fields_2015	
	eichorn_bcell_2014	B_cell
	gonzalez_2014_mmu	Brain
	cho_2015	
	laguesse_2015	
	deklerck_2015	C2C12
	ingolia_2014_mmu	E14
	Ingolia_2011	
	ingolia_2011	
	Mouse gliomal cells	Glioma
	Mouse liver cell	Liver
	eichorn_liver_2014	
	gao_liver_2014	
	gerashchenko_2016	
	janich_2015	
	Mouse Embryonic Fibroblast (MEFs)	MEF
	thoreen_2012	
	lee_2012_mmu	

gao_mef_2014	
reid_er_2016	
reid_cytosol_2016	
reid_2014	
Mouse Embryonic Stem Cells	MESC
katz_2014	NSC
guo_2010_mmu	Neutrophil
you_2015	R1E
blanco_2016	Skin_tumor
diaz_munoz_2015	Spleen_B_cell
castaneda_2014	Testis
hurt_2013	v6-5

Supp. Table S4 | MetamORF cell types and ontologies

MetamORF cell type	Ontology terms*									
	**CL**	**CLO**	**BTO**	**HCAO**	**FMA**	**OBI**	**NCIT**	**EFO**	**BAO**	**OMIT**
HCT116			BTO:0001109					EFO:0002824	CLO:0003665	OMIT:0023581
THP-1			BTO:0001370					EFO:0001253	CLO:0009348	
HEK293		CLO:0001230	BTO:0000007					EFO:0001182		OMIT:0027010
NCCIT		CLO:0007955	BTO:0004180							
HeLa			BTO:0000567				NCIT:C20226	EFO:0001185	CLO:0003684	OMIT:0007538
HEK293T			BTO:0002181					EFO:0001184		
Brain	UBERON:0000955	UBERON:0000955	BTO:0000142	UBERON:0000955	FMA:50801	UBERON:0000955	NCIT:C12439	UBERON:0000955	UBERON:0000955	OMIT:0003277
HFF	CL:1001608	CLO:0000556	BTO:0002245							
MDA-MB-231			BTO:0000815					EFO:0001209	CLO:0007634	
BJ		CLO:0001980	BTO:0003807					EFO:0002779	BAO:0002670	
MM1S		CLO:0037203						EFO:0005724		
U2OS			BTO:0001938					EFO:0002869	CLO:0009454	
Jurkat		BTO:0000661	BTO:0000661			OBI:1110035		EFO:0002796	CLO:0007043	OMIT:0019249
RPE-1	CL:0002586	BTO:0002334					NCIT:C33470			
Skeletal_muscle	CL:0000188		BTO:0004392				NCIT:C48687			
hES		CLO:0037280	BTO:0001581							OMIT:0001087
Neutrophil	CL:0000775		BTO:0000130				NCIT:C12533			
v6-5								EFO:0006308		
E14			BTO:0005136					EFO:0007075		
NSC	CL:0000047	CLO:0000051								
MEF	CL:2000042		BTO:0002572				NCIT:C24196	EFO:0004040		

Spleen_B_cell	CL:0000236		BTO:0000776				NCIT:C12474			OMIT:0016721
3T3		CLO:0001345								OMIT:0016968
B_cell	CL:0000236		BTO:0000776	CL:0000236			NCIT:C12474			OMIT:0002778
Liver		EFO:0000887	BTO:0000759	UBERON:0002107	FMA:63179	UBERON:0002107	NCIT:C12392	UBERON:0002107	UBERON:0002107	OMIT:0009182
BMDC			BTO:0003857				NCIT:C156591			
Skin_tumor									DOID:3178	
Testis	UBERON:0000473	UBERON:0000473	BTO:0001363	UBERON:0000473	FMA:7210	UBERON:0000473	NCIT:C12412	EFO:0000984	UBERON:0000473	OMIT:0014592
C2C12			BTO:0000165					EFO:0001098	BAO:0002708	
R1E		CLO:0008700	BTO:0004500					EFO:0002076		
HAP1								EFO:0007598		OMIT:0037111
Blood	CL:0000081	EFO:0000296	BTO:0000089	CL:0000081	FMA:62844	UBERON:0000178		CL:0000081	UBERON:0000178	OMIT:0003133
Monocyte	CL:0000576	CL:0000576	BTO:0000876	CL:0000576	FMA:62864		NCIT:C12547	CL:0000576		
LCL			BTO:0003335					EFO:0005292		
MCF7			BTO:0000093				NCIT:C18096	EFO:0001203	CLO:0007606	OMIT:0028025
MCF10A		CLO:0007599	BTO:0001939					EFO:0001200		
Flp-In_T-REx-293		CLO:0037238	BTO:0006149							
Brain_tumor		DOID:1319	BTO:0001573				NCIT:C2907	MONDO:0001657	DOID:1319	OMIT:0003288
MESC	CL:0002322	CLO:0037317	BTO:0001581		FMA:82841		NCIT:C12935	EFO:0004038		OMIT:0001088
Glioma		EFO:0000520	BTO:0000526				NCIT:C3059	EFO:0005543	DOID:0060108	OMIT:0007103
Breast	UBERON:0000310	UBERON:0000310	BTO:0000149	UBERON:0000310	FMA:19898	UBERON:0000310	NCIT:C12971	UBERON:0000310	UBERON:0000310	OMIT:0003296

*** Some ontology terms may refer themselves to external ontologies**

Supp. Table S5 | Kozak contexts definitions. The start codon contains the nucleotides +1 to +3. The same patterns with variation allowed on the nucleotides between +1 to +3 position were used to compute the Kozak contexts of sORFs with alternative start codons.

	**-6**	**-5**	**-4**	**-3**	**-2**	**-1**	**+1**	**+2**	**+3**	**+4**
Optimal	G	C	C	R	C	C	**A**	**T**	**G**	G
Strong	N	N	N	R	N	N	**A**	**T**	**G**	G
Moderate	N	N	N	R	N	N	**A**	**T**	**G**	A / T / C
or	N	N	N	Y	N	N	**A**	**T**	**G**	G
Weak	N	N	N	Y	N	N	**A**	**T**	**G**	A / T / C

R = A / G (purine), Y = C / T (pyrimidine)

Supp. Table S6 | Regular expressions corresponding to Kozak contexts. The Kozak contexts have been computed using the criteria described in the Supp. table S2. To perform this computation, regular expressions have been searched in the sequences flanking the ORF start codons.

**Kozak context**	**Regular expression**
Optimal	`GCC[AG]CC.{3}G`
Strong	`.{3}[AG].{2}.{3}G`
Moderate	`(.{3}[AG].{2}.{3}[ATC]|.{3}[CT].{2}.{3}G)`
Weak	`.{3}[CT].{2}.{3}[ACT]`

Supp. Table S7 | Source of the gene lists used to perform the enrichment analysis

**Gene list**	**Source**	**Description**
ATF4 targets[1]	Han *et al.*, 2013, Nat. Cell. Biol.	Table S1: "Supplementary Table S2. List of ATF4 and CHOP target genes that have binding peaks within 3kb from TSS of annotated gene." restricted to ATF4 targets (i.e. genes with "Overlap=Common" or "ATF4_Only")
CHOP targets[2]	Han *et al.*, 2013, Nat. Cell. Biol.	Table S1: "Supplementary Table S2. List of ATF4 and CHOP target genes that have binding peaks within 3kb from TSS of annotated gene." restricted to ATF4 targets (i.e. genes with "Overlap=Common" or "CHOP_Only")
Genes congruently up-regulated[3]	Guan *et al.*, 2017, Mol. Cell.	Get upon request - Congruent (Transcriptional and translational) up-regulation at 16h (chronic ER stress)
Genes transitionally up-regulated[4]	Guan *et al.*, 2017, Mol. Cell.	Get on request - Translational up-regulation at 1h (accute ER stress)

Universe	Gene ontology / gProfiler	All protein coding genes with at least one gene ontology annotation have been included in the universe. The lists of GO terms associated with their Ensembl gene IDs have been downloaded using the gProfiler web interface as a gmt file (data sources tab).

Supp. Table S8 | Comparison of MetamORF with existing sORF-related databases

REFERENCES

1. Andreev,D.E., Arnold,M., Kiniry,S.J., Loughran,G., Michel,A.M., Rachinskii,D. and Baranov,P.V. (2018) TASEP modelling provides a parsimonious explanation for the ability of a single uORF to derepress translation during the integrated stress response. *eLife*, **7**.

2. Rodriguez,C.M., Chun,S.Y., Mills,R.E. and Todd,P.K. (2019) Translation of upstream open reading frames in a model of neuronal differentiation. *BMC Genomics*, **20**, 391.

3. Sharipov,R.N., Yevshin,I.S., Kondrakhin,Y.V. and Volkova,O.A. (2014) RiboSeqDB – a repository of selected human and mouse ribosome footprint and RNA-seq data. *Virtual Biology*, **1**, 37-46–46.

4. Evans,V.C., Barker,G., Heesom,K.J., Fan,J., Bessant,C. and Matthews,D.A. (2012) De novo derivation of proteomes from transcriptomes for transcript and protein identification. *Nat. Methods*, **9**, 1207–1211.

5. Chew,G.-L., Pauli,A. and Schier,A.F. (2016) Conservation of uORF repressiveness and sequence features in mouse, human and zebrafish. *Nat. Commun.*, **7**, 11663.

6. Wethmar,K., Barbosa-Silva,A., Andrade-Navarro,M.A. and Leutz,A. (2014) uORFdb--a comprehensive literature database on eukaryotic uORF biology. *Nucleic Acids Res.*, **42**, D60-67.

7. Fields,A.P., Rodriguez,E.H., Jovanovic,M., Stern-Ginossar,N., Haas,B.J., Mertins,P., Raychowdhury,R., Hacohen,N., Carr,S.A., Ingolia,N.T., *et al.* (2015) A regression-based analysis of ribosome-profiling data reveals a conserved complexity to mammalian translation. *Mol. Cell.*, **60**, 816–827.

8. Liu,W., Xiang,L., Zheng,T., Jin,J. and Zhang,G. (2018) TranslatomeDB: a comprehensive database and cloud-based analysis platform for translatome sequencing data. *Nucleic Acids Res.*, **46**, D206–D212.

9. Wang,H., Yang,L., Wang,Y., Chen,L., Li,H. and Xie,Z. (2019) RPFdb v2.0: an updated database for genome-wide information of translated mRNA generated from ribosome profiling. *Nucleic Acids Res.*, **47**, D230–D234.

10. Hao,Y., Zhang,L., Niu,Y., Cai,T., Luo,J., He,S., Zhang,B., Zhang,D., Qin,Y., Yang,F., *et al.* (2018) SmProt: a database of small proteins encoded by annotated coding and non-coding RNA loci. *Brief. Bioinform.*, **19**, 636–643.

11. Lee,S., Liu,B., Lee,S., Huang,S.-X., Shen,B. and Qian,S.-B. (2012) Global mapping of translation initiation sites in mammalian cells at single-nucleotide resolution. *Proc. Natl. Acad. Sci. U.S.A.*, **109**, E2424-2432.

12. McGillivray,P., Ault,R., Pawashe,M., Kitchen,R., Balasubramanian,S. and Gerstein,M. (2018) A comprehensive catalog of predicted functional upstream open reading frames in humans. *Nucleic Acids Res.*, **46**, 3326–3338.

13. Mackowiak,S.D., Zauber,H., Bielow,C., Thiel,D., Kutz,K., Calviello,L., Mastrobuoni,G., Rajewsky,N., Kempa,S., Selbach,M., *et al.* (2015) Extensive identification and analysis of conserved small ORFs in animals. *Genome Biol.*, **16**.

14. Erhard,F., Halenius,A., Zimmermann,C., L'Hernault,A., Kowalewski,D.J., Weekes,M.P., Stevanovic,S., Zimmer,R. and Dölken,L. (2018) Improved Ribo-seq enables identification of cryptic translation events. *Nat. Methods*, **15**, 363–366.

15. Johnstone,T.G., Bazzini,A.A. and Giraldez,A.J. (2016) Upstream ORFs are prevalent translational repressors in vertebrates. *EMBO J.*, **35**, 706–723.

16. Laumont,C.M., Daouda,T., Laverdure,J.-P., Bonneil,É., Caron-Lizotte,O., Hardy,M.-P., Granados,D.P., Durette,C., Lemieux,S., Thibault,P., *et al.* (2016) Global proteogenomic analysis of human MHC class I-associated peptides derived from non-canonical reading frames. *Nat. Commun.*, **7**, 10238.

17. Samandi,S., Roy,A.V., Delcourt,V., Lucier,J.-F., Gagnon,J., Beaudoin,M.C., Vanderperre,B., Breton,M.-A., Motard,J., Jacques,J.-F., *et al.* (2017) Deep transcriptome annotation enables the discovery and functional characterization of cryptic small proteins. *eLife*, **6**.

18. Olexiouk,V., Van Criekinge,W. and Menschaert,G. (2018) An update on sORFs.org: a repository of small ORFs identified by ribosome profiling. *Nucleic Acids Res.*, **46**, D497–D502.

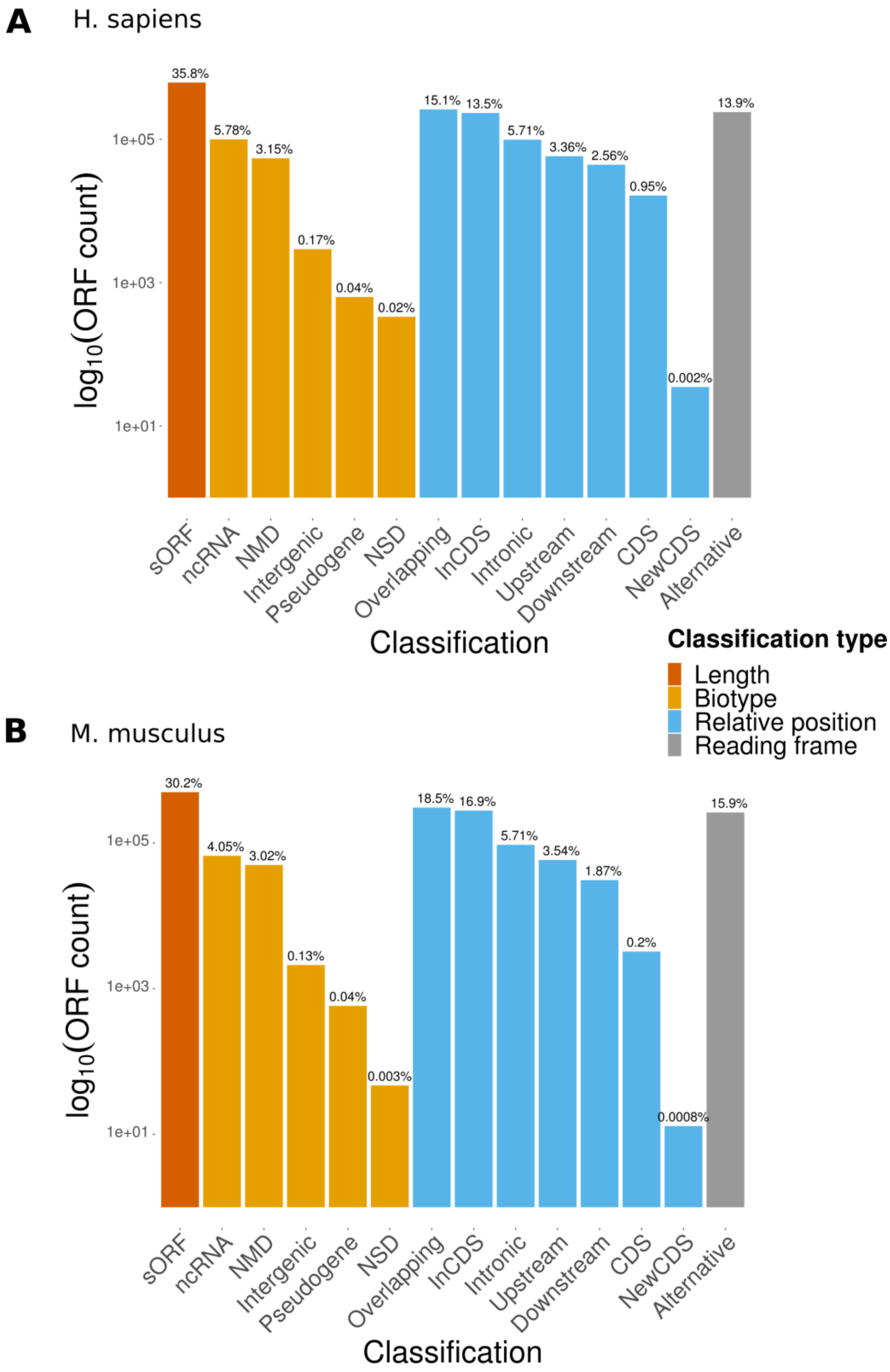

Supp. Figure S1 | Count of ORFs in each class. The barplot represent the count of ORFs annotated for each class for (A) *H. sapiens* and (B) *M.* musculus. The percentages displayed over the bars indicates the proportion of ORFs annotated in the class over the total number of annotations computed by the MetamORF workflow for the species.

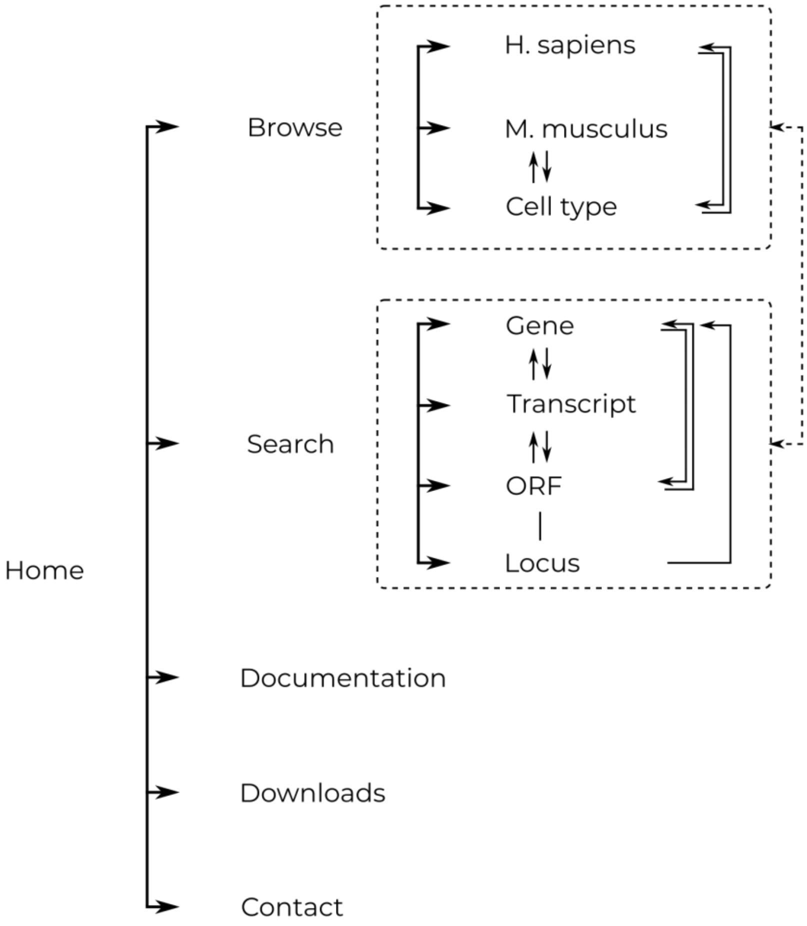

Supp. Figure S2 | Relational map of MetamORF web interface.
